# LIFETIME REVICTIMIZATION: EVIDENCE FROM THE WISCONSIN LONGITUDINAL STUDY

**DOI:** 10.1093/geroni/igad104.0138

**Published:** 2023-12-21

**Authors:** Jooyoung Kong, Scott Easton, Jason Fletcher

**Affiliations:** University of Wisconsin-Madison, Madison, Wisconsin, United States; Boston College, Boston, Massachusetts, United States; University of Wisconsin-Madison, Madison, Wisconsin, United States

## Abstract

Research has documented the increasing prevalence of elder abuse victimization and its devastating impact on the health and well-being of older adults. However, a life-course view of the victimization experiences of older adults has rarely been adopted in the elder abuse literature. The current study examined lifetime links between victimization experiences that span childhood, adulthood, and older adulthood. Specifically, we examined: a) the mediating effect of adverse childhood experiences (ACE) on elder abuse victimization via intimate partner violence (IPV) victimization in middle adulthood, and b) whether this indirect association differs by gender. Data were obtained from the Wisconsin Longitudinal Study (WLS). We analyzed the previous and current victimization experiences of a total of 5,391 older adults in their early 70s and estimated the mediational and moderated mediation models. The key results indicated that a higher ACE score was associated with exposure to IPV victimization in middle adulthood, which was in turn associated with exposure to elder abuse victimization. This indirect association was stronger for women than for men. Regarding specific types of childhood victimization, parental physical abuse, sexual abuse, and witnessing domestic violence significantly predicted elder abuse victimization via IPV. Our results support the phenomenon of lifetime victimization, whereby an individual experiences reoccurring forms of victimization across the life span from childhood to late adulthood. Findings highlight the compelling need for the assessment of cumulative victimization experiences and their impact on elder abuse victims. A life course-based, trauma-informed approach would greatly enhance prevention and intervention services for elder abuse.

